# A Solitary Seborrheic Keratosis Mimicking Malignant Melanoma

**DOI:** 10.7759/cureus.107339

**Published:** 2026-04-19

**Authors:** Hanna Mass, Shyam S Raghavan, Morteza Khodaee

**Affiliations:** 1 Internal Medicine, University of Colorado School of Medicine, Aurora, USA; 2 Pathology, University of Colorado School of Medicine, Aurora, USA; 3 Sports Medicine, University of Colorado School of Medicine, Aurora, USA

**Keywords:** dermoscopy, excisional biopsy, hyperpigmented plaques, melanoma, stuck on appearance

## Abstract

Large hyperpigmented skin lesions are a common presentation in primary care settings. Differential diagnosis includes several benign and malignant lesions, including seborrheic keratosis and malignant melanoma; thus, excisional biopsy should be performed if there is any uncertainty. We present a man in his 60s with a painless, slow-growing dark brown plaque on his upper back. Due to the size and irregularity of the lesion, as well as the patient’s dermatological preference, an excisional biopsy was performed, which confirmed the diagnosis of seborrheic keratosis.

## Introduction

Large hyperpigmented skin lesions are a common presentation in older adults in primary care settings. The majority of cases are benign, and diagnosis can be made clinically [1]. However, lesions with irregular or atypical features warrant broader consideration of the differential diagnosis to ensure that rare malignant etiologies are not overlooked [1]. Benign conditions that may present as large hyperpigmented lesions include seborrheic keratoses, solar lentigines, melanocyte nevi, acanthosis nigricans, and verruca vulgaris [1,2]. In sun-exposed areas, pigmented actinic keratosis and keratinocyte carcinoma should also be considered, though these typically lack the classic stuck-on appearance [1]. While less common, malignant melanoma and carcinomas have been reported to mimic clinical features of seborrheic keratosis [1]. Therefore, despite the differential diagnosis most often favoring benign lesions, any irregularity in presentation or diagnostic uncertainty should prompt biopsy for definitive diagnosis. In this case, we present a man with a large, irregular, hyperpigmented skin lesion on his back, which had existed for several years.

## Case presentation

**Figure 1:**
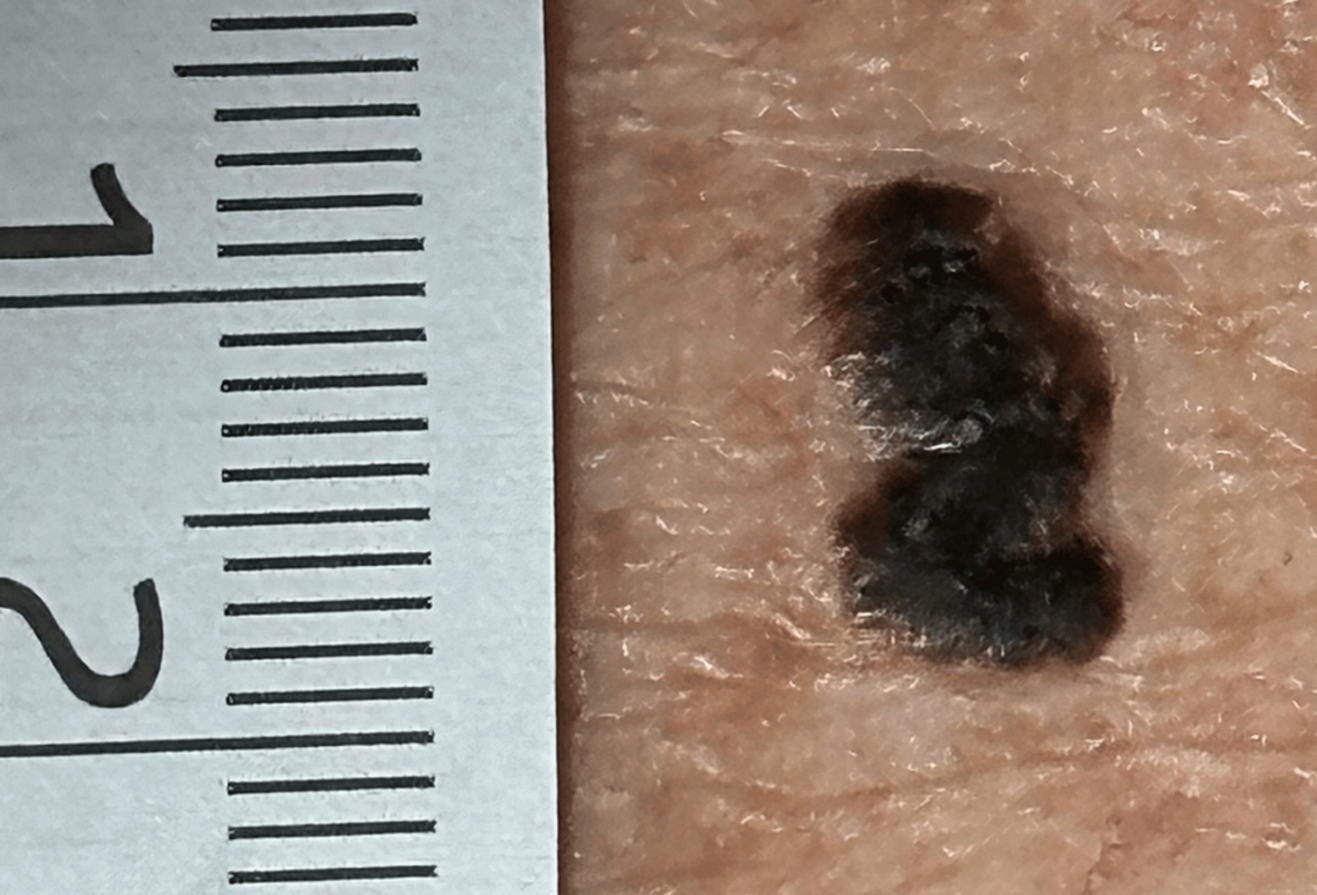
A non-tender 1.2 x 0.7 cm, irregularly oval-shaped, brown-black plaque on the left upper back.

A man in his 60s presented with a dark brown skin lesion on his upper back. The lesion was painless and had been slowly growing over the last several years. On physical examination, there was a non-tender, mildly raised, dark, brown-colored plaque on the left upper back (Figure 1).

**Figure 2:**
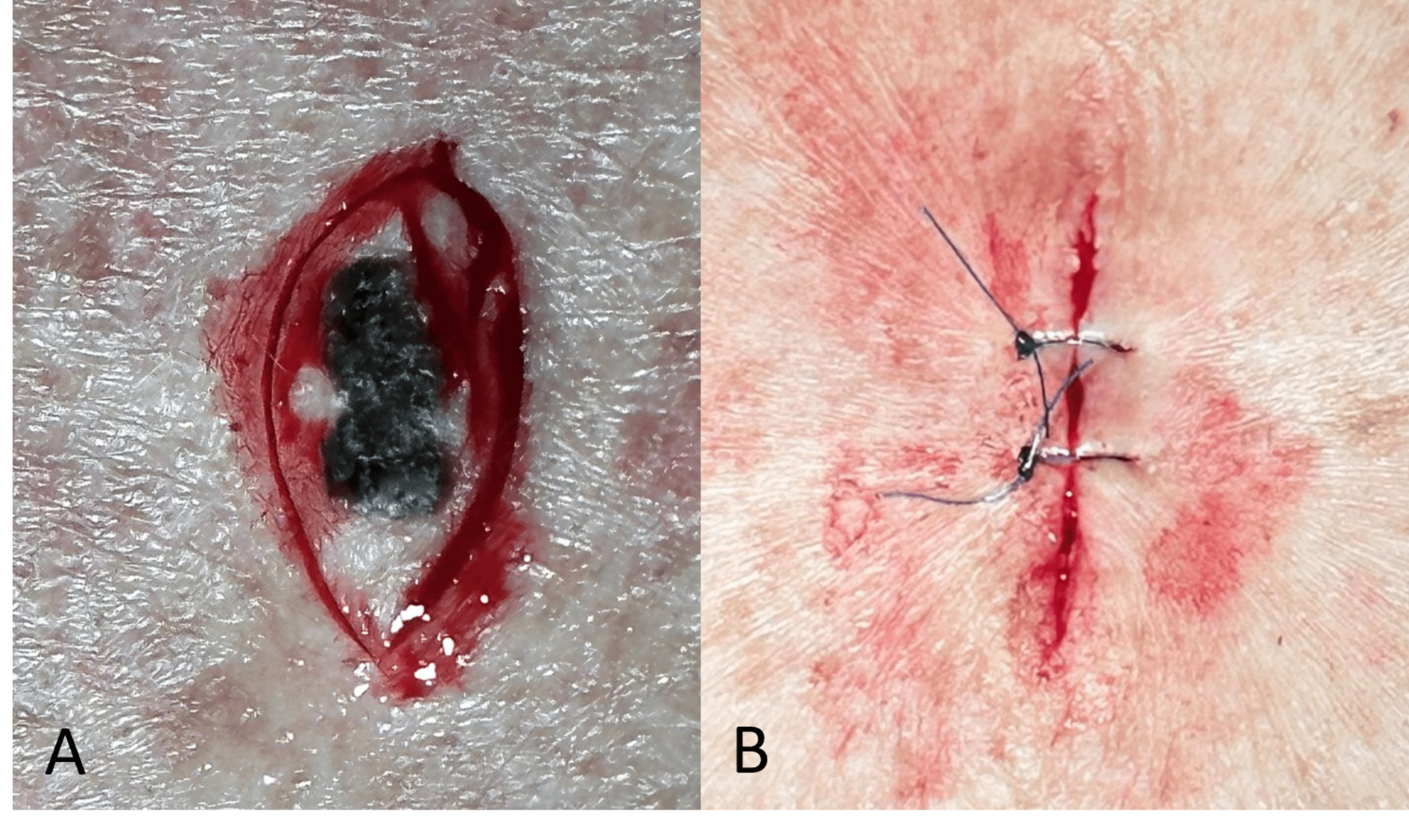
An elliptical incision was made to excise the lesion (A). The wound was subsequently closed using interrupted sutures (B).

Differential diagnosis included seborrheic keratosis and malignant melanoma. After discussing options with the patient, he elected to have the lesion removed. Excisional biopsy was performed, and the sample was sent for pathology (Figure 2).

**Figure 3:**
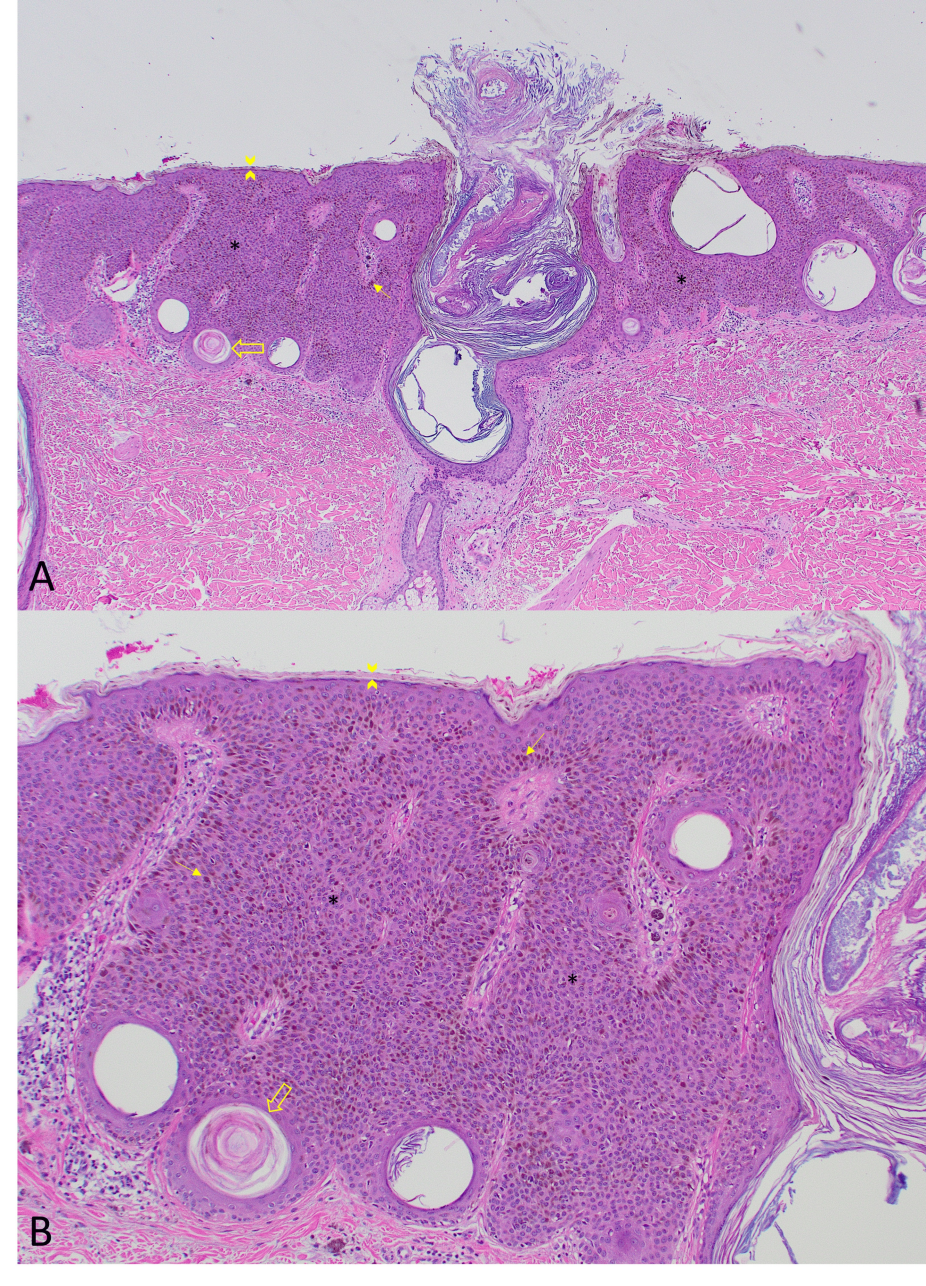
Hematoxylin and eosin stain sections at 40x magnification (A) and at 100x magnification (B). Sections show an epidermal proliferation of monotonous cuboidal keratinocytes (*) with horn cysts (open arrows) and abundant pigmented parakeratosis (arrowheads). There is increased pigmentation to the keratinocytes (arrows) without evidence of a melanocytic proliferation.

Histologic sections of the excision specimen demonstrated an acanthotic epidermal proliferation of small, bland cuboidal keratinocytes with hyperkeratosis and horn pseudocysts (Figure 3). There was no significant cytologic atypia or mitotic activity. There was a marked increase in keratinocyte pigmentation without evidence of a melanocytic proliferation. There was abundant overlying pigmented parakeratosis, which can be appreciated secondary to irritation. These findings are diagnostic of a pigmented seborrheic keratosis. The patient followed up 13 days later for suture removal, with normal healing and no complications noted.

## Discussion

Seborrheic keratoses are very common benign skin tumors composed of immature epidermal keratinocytes [1,2]. Though their pathogenesis is not well understood, factors including older age, genetic predisposition, and exposure to ultraviolet radiation may increase the risk for development [2,3]. The prevalence of seborrheic keratosis increases with older age, reaching nearly 100 percent in individuals over the age of 60 [1,3,4]. Clinically, seborrheic keratosis typically presents in middle-aged and older adults as round, well-demarcated, brown to black lesions [2]. They characteristically exhibit a stuck-on appearance with a dull, waxy, verrucous surface and grow gradually over several years [2]. This unique stuck-on appearance of a raised plaque is a key feature in distinguishing seborrheic keratosis from other hyperpigmented lesions [2]. Lesions most commonly occur on the face and upper trunk but may arise anywhere on the body except the palms, soles, and mucous membranes [2,3].

Diagnosis of seborrheic keratosis is typically made clinically based on its distinctive appearance. Additionally, the use of dermoscopy, a non-invasive tool, can enhance the accuracy of diagnosis by allowing for a magnified view of the subsurface structures [3,5]. Key features of seborrheic keratosis identified with dermoscopy include multiple milia-like cysts, comedo-like openings, fissures/ridges, and sharp demarcation [1,3,5]. Pigment networks or globules, which are features of melanocytic lesions, should be absent [1,2]. However, it is important to note that access to dermoscopy may be limited in rural or resource-constrained primary care settings.

The presence of ulcerations, rapid growth, asymmetry, border irregularity, color variegation, and a diameter greater than 6mm should raise concern for malignancy [2,6]. In such cases, excisional biopsy is recommended as melanoma with features mimicking seborrheic keratosis may be difficult to distinguish [2,6].

Histopathologically, pigmented seborrheic keratosis is characterized by a monoclonal proliferation of keratinocytes, along with marked basal layer pigmentation, papillomatosis, acanthosis, and hyperkeratosis [7-9].

While treatment is not medically indicated for benign seborrheic keratoses, lesions may be removed for cosmetic reason or patient preference if they are symptomatic [2,9]. Providers and patients should engage in shared decision-making, considering the risks and benefits of various locally destructive treatments or topical agents.

## Conclusions

Seborrheic keratoses are common skin lesions, particularly in the geriatric population, and diagnosis can be made clinically in the majority of cases. When available, dermoscopy is a useful tool in differentiating between benign and malignant cases. Large hyperpigmented plaques are usually benign (e.g., seborrheic keratosis); however, an excisional biopsy should be performed, particularly in cases with any concern for malignant melanoma.
